# Multiomics analyses identified epigenetic modulation of the S100A gene family in Kawasaki disease and their significant involvement in neutrophil transendothelial migration

**DOI:** 10.1186/s13148-018-0557-1

**Published:** 2018-11-01

**Authors:** Lien-Hung Huang, Ho-Chang Kuo, Cheng-Tsung Pan, Yeong-Shin Lin, Ying-Hsien Huang, Sung-Chou Li

**Affiliations:** 1 Genomics and Proteomics Core Laboratory, Department of Medical Research, Kaohsiung Chang Gung Memorial Hospital, 12th Floor, Children’s Hospital, No.123, Dapi Rd, Niaosong District, Kaohsiung, 83301 Taiwan; 2 Department of Pediatrics, Kaohsiung Chang Gung Memorial Hospital and Chang Gung University College of Medicine, Kaohsiung, Taiwan; 3 Kawasaki Disease Center, Kaohsiung Chang Gung Memorial Hospital, Kaohsiung, Taiwan; 4 Institute of Bioinformatics and Systems Biology, National Chiao Tung University, Hsinchu, Taiwan; 5 Department of Biological Science and Technology, National Chiao Tung University, Hsinchu, Taiwan

**Keywords:** Kawasaki disease, DNA methylation, CpG marker, Gene expression, Correlation, S100A gene family, Leukocyte transendothelial migration

## Abstract

**Background:**

Kawasaki disease (KD) is a prevalent pediatric disease worldwide and can cause coronary artery aneurysm as a severe complication. Typically, DNA methylation is thought to repress the expression of nearby genes. However, the cases in which DNA methylation promotes gene expression have been reported. In addition, globally, to what extent DNA methylation affects gene expression and how it contributes to the pathogenesis of KD are not yet well understood.

**Methods:**

To address these important biological questions, we enrolled subjects, collected DNA and RNA samples from the subjects’ total white blood cells, and performed DNA methylation (M450K) and gene expression (HTA 2.0) microarray assays.

**Results:**

By analyzing the variation ratios of CpG beta values (methylation percentage) and gene expression intensities, we first concluded that the CpG markers close (− 1500 bp to + 500 bp) to the transcription start sites had higher variation ratios, reflecting significant regulation capacities. Next, we observed that, globally speaking, gene expression was modestly negatively correlated (correlation rho ≈ − 0.2) with the DNA methylation status of both upstream and downstream CpG markers in the promoter region. Third, we found that specific CpG markers were hypo-methylated in disease samples compared with healthy samples and hyper-methylated in convalescent samples compared with disease samples, promoting and repressing S100A genes’ expressions, respectively. Finally, using an in vitro cell model, we demonstrated that S100A family proteins enhanced leukocyte transendothelial migration in KD.

**Conclusions:**

This is the first study to integrate genome-wide DNA methylation with gene expression assays in KD and showed that the S100A family plays important roles in the pathogenesis of KD.

**Electronic supplementary material:**

The online version of this article (10.1186/s13148-018-0557-1) contains supplementary material, which is available to authorized users.

## Background

DNA methylation is a cellular activity at which the hydrogen atom on carbon 5 in the cytosine of CpG di-nucleotide (also called CpG marker) is replaced by a methyl group [1]. Through DNA methylation, gene activity can be silenced either by interfering with the binding of transcription factors or by interacting with the modification of histone protein [2].

Previous studies have demonstrated that abnormal DNA methylation led to gastric carcinogenesis by either hyper-methylating several tumor-suppressive miRNAs [3–5] or hypo-methylating onco-miR [6]. In addition, DNA methylation also regulated the erythropoiesis of embryonic stem cell [7], the pathogenesis of idiopathic pulmonary fibrosis [8], the neurodevelopment of the human hippocampus [9], and other processes. In addition to regulating disease pathogenesis, DNA methylation also performs long-term regulatory activities. Children suffered from early adversity, such as being raised in an orphanage, had higher global methylation patterns, and their neural-related genes were silenced by hyper-methylation [10]. Moreover, DNA methylation was also involved in nutritional control of the reproductive statuses of honeybees, as a result controlling the generation of workers or queens [11]. Through regulating the expressions of many critical genes, DNA methylation plays important roles not only in cellular activities but also in many human diseases. However, few DNA methylation-related studies have been conducted for Kawasaki disease.

Kawasaki disease (KD) is an acute systemic vasculitis disease, and it usually attacks children less than 5 years of age. The most severe complication of KD is coronary artery aneurysm (CAA), which affects approximately 20–25% of KD patients without timely treatment with intravenous immunoglobulin (IVIG) [12]. Therefore, KD is the major cause of acquired heart disease in children in developed countries [13]. The etiopathogenesis of KD may be attributed to the combined effects of genetics, immunity, and infection [14]. Although the exact etiology of KD is still unknown, predicting KD is possible with molecular markers [15]. To date, only few studies have focused on the regulation of DNA methylation in KD [16, 17]. However, these studies only conducted profiling of DNA methylation patterns, without further investigating whether the extent of DNA methylation affected the pathogenesis of KD. In addition, although considered to be negatively correlated with gene expression, DNA methylation of several CpG markers was reported to promote gene expressions [18, 19].

To answer these questions, we conducted a study in which we collected DNA and RNA samples from KD subjects, followed by combining the DNA methylation profiling data with the gene expression information for a systems biology perspective. Previous studies determined the correlations between DNA methylation and gene expression with CpG beta values (methylation percentages) and gene expression intensities [19]. In this study, we focused on the variation ratios of CpG beta values and the ones of gene expression intensities among different sets of samples. First, we identified modestly negative correlations between DNA methylation and gene expression regardless of whether the CpG markers were located upstream or downstream of the promoter regions. Second, we showed that the S100A gene family enhanced leukocyte transendothelial migration in KD with an in vitro cell model.

## Results

### Subject information

In this study, we enrolled 24 non-fever healthy control subjects (HC), 21 fever control subjects (FC, patients with fever but not diagnosed as KD or not having a KD history) and 18 KD patients. Blood samples from the KD patients were drawn both at the acute phase 1 day before IVIG treatment (KD1) and at the convalescent phase 3 weeks after IVIG treatment (KD3). Blood samples from the remaining subjects were drawn once. As shown in Additional file 1, 8 out of the 21 FC subjects suffered from acute sinusitis and 19.5 and 14.3% of the FC subject population had gastroenteritis and bronchopneumonia, respectively. No significant difference was observed in age (*p* = 0.0536, *t* test) or gender (*p* = 1, Fisher’s exact test) between the 12 HC and 12 KD subjects whose samples were used for the Illumina HumanMethylation 450 BeadChip assays (M450 K). In addition, no significant difference was observed in age (*p* = 0.1108, *t* test) or gender (*p* = 0.7, Fisher’s exact test) between the 18 HC and 18 KD subjects used for the Affymetrix GeneChip® Human Transcriptome Array 2.0 (HTA 2.0) assays. All of the KD patients met the diagnosis criteria of AHA 2004 [20].

### DNA methylation variations among samples

**Fig. 1:**
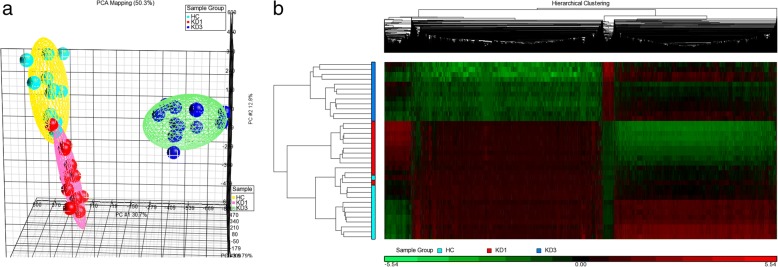
DNA methylation profiles among the HC, KD1, and KD3 sets. We conducted methylation microarray (M450K) assays on 12 HC, 12 KD1, and 12 KD3 samples. The generated raw data was analyzed with Partek to produce **a** a PCA plot and **b** a heat map. The heat map was plotted with the methylation profiles of 25,984 CpG markers

From the total HC, KD1, and KD3 DNA samples, we selected 12 HC, 12 KD1, and 12 KD3 ones for bisulfite conversion, followed by M450K assays on the 36 bisulfite converted DNA samples (Additional file 1). The generated raw data was analyzed with Partek. First, we examined the overall methylation patterns of the three sets using a PCA plot. As shown in Fig. 1a, the three sets can be clearly distinguished in terms of their methylation patterns. The KD3 set was located distinct from the other two ones, whereas, the HC and KD1 sets slightly overlapped with each other. When the FDR < 0.05 and variation ratio > 1.1 criteria were specified, there were 12,209, 13,936, and 14,643 significant CpG markers among the KD1 vs. HC, KD3 vs. HC, and KD3 vs. KD1 comparisons (Table 1), respectively. These significant CpG markers formed a union of 25,984 CpG markers, and the heat map of which is demonstrated in Fig. 1b. Table 1 and Fig. 1b show that most of the significant CpG markers in the KD1 vs. HC comparison were hypo-methylated in the KD1 samples, reflecting hypo-methylation of CpG markers with the onset of KD.

**Table 1:** Summary of significant CpG markers among the comparisons

Comparison	# all markers	# hyper markers	# hypo markers
KD1 vs. HC	12,209	1484	10,725
KD3 vs. HC	13,936	505	13,431
KD3 vs. KD1	14,643	4669	9974

Based on the criteria of an FDR < 0.05 and variation ratio > 1.1, we identified significant CpG markers among the three comparisons. Hyper marker and hypo marker denoted the significant hyper-methylated and hypo-methylated markers, respectively

The Manhattan plots of the three comparisons were also provided (Additional files 2, 3, and 4). Although the numbers of significant CpG markers in the three comparisons were almost equivalent (Table 1), the Manhattan plots showed that the KD3 vs. HC and KD3 vs. KD1 comparisons, both of which involved in the IVIG administration factor, had much lower *p* values and much more significant CpG markers. In our previous study, using M27K assays, we observed that IVIG administration had a much stronger impact on methylation variation than disease onset did [16]. Our current data also supported this finding.

### Methylation variations of CpG markers within the putative promoter regions

**Fig. 2:**
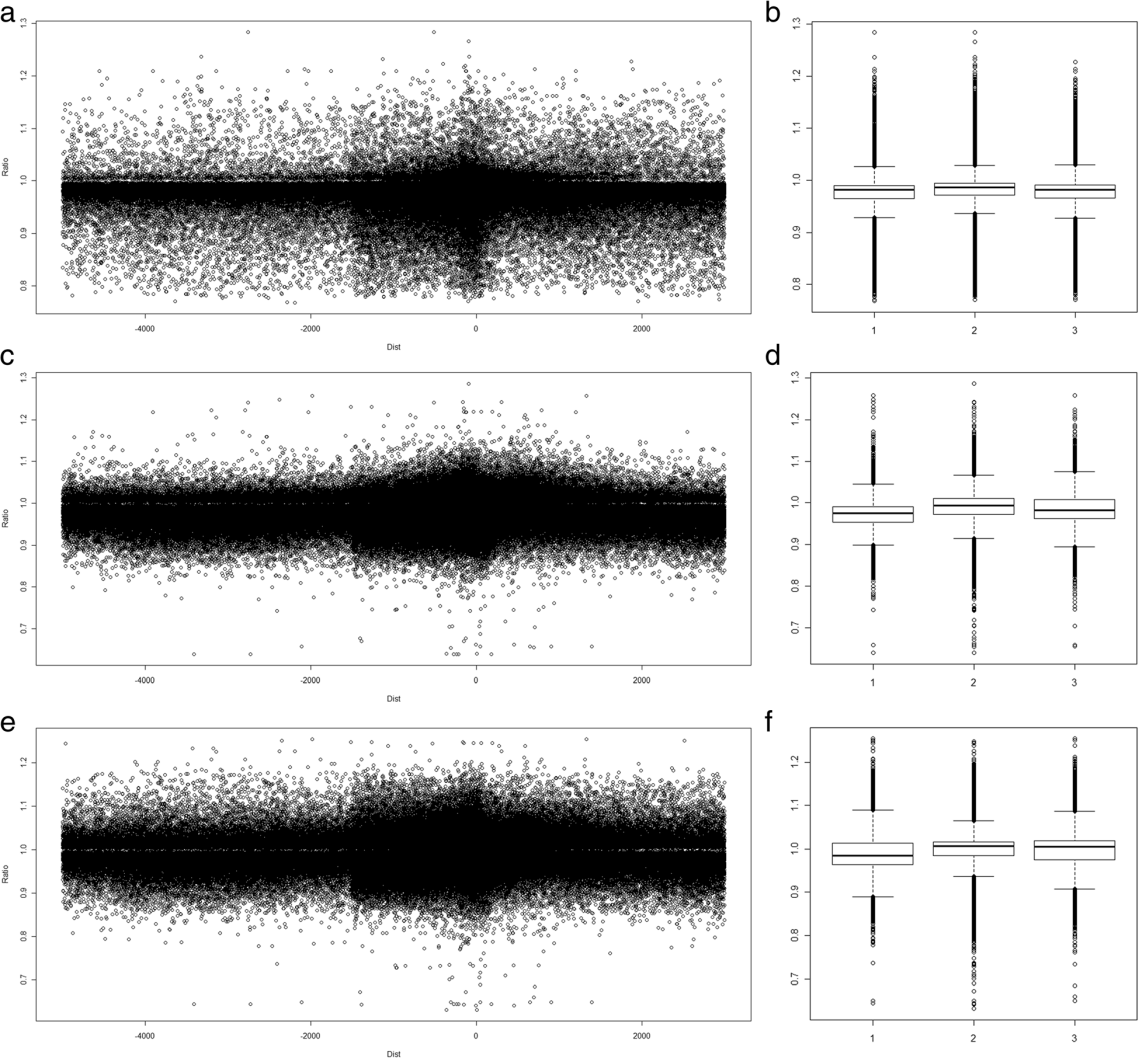
Methylation variations of significant CpG markers within the putative promoter regions. By referring to the RefSeq 41 annotation, we can determine a CpG marker’s distances to transcription start site (TSS) of a gene’s transcript. Then, we can also determine the relative locations of CpG markers within the putative promoter regions, which are the genomic regions ranging from − 5000 bp to + 3000 bp of a transcript’s TSS. **a**, **c**, **e** For each CpG marker, the *X* and *Y* axes denoted its distance to TSS and its methylation variation, respectively. Using the two arrows, the promoter was split into three sub-regions, the left, the core and the right sub-regions. The methylation variations (average ± S.D.) of the CpG markers located within each sub-region were labeled. The sample sizes for sub-figures **a**, **c**, **e** were 205,306, 393,023, and 385,840, respectively. **b**, **d**, **f** The box plots and *t* test demonstrated that the CpG markers within the core sub-region varied more than those within the other two sub-regions (*P* < 2.2E−16 for the six comparisons)

Next, we investigated the methylation variations of CpG markers based on the distance to the transcription start sites (TSSs) of genes. Since a promoter is a rough and ambiguous region relative to the TSS of a gene, studies have defined their putative promoter regions with different distances to the TSS [21, 22]. In this study, we adopted the default parameter of Partek and defined a promoter as the region ranging from − 5000 to 3000 of a transcript’s TSS (RefSeq 41 annotation). Then, we mapped all significant CpG makers (*P* < 0.05) back to the promoters and marked their methylation variation ratios. According to Fig. 2, the densities of the significant CpG markers seemed to be higher within the − 1500 to 500 regions than the ones out of this region. To examine the densities of CpG markers within the promoters, we also mapped all CpG markers (both significant and non-significant) back to the promoters. As a result, we observed results similar to those shown in Fig. 2 (Additional file 5). Therefore, higher densities of CpG makers within the − 1500 to 500 regions were an intrinsic characteristic of the M450K microarray chip.

Figure 2a, c, e also shows that CpG markers within the − 1500 to 500 region tended to vary more than the rest CpG markers outside of this region. To investigate this issue, we divided the putative promoter region (− 5000 to 3000 bp) into three sub-regions as follows: the left (− 5000 to − 1500 bp), core (− 1500 to 500 bp), and right (500 to 3000 bp) sub-regions. As shown in Fig. 2b, d, f, consistently among the three comparisons, the CpG markers within the core regions significantly varied more than the ones within the two adjacent regions (*P* < 2.2E−16), implying that the CpG makers closer to the TSS of the transcript regulated gene expression more significantly.

### Gene expression variations among samples

**Fig. 3:**
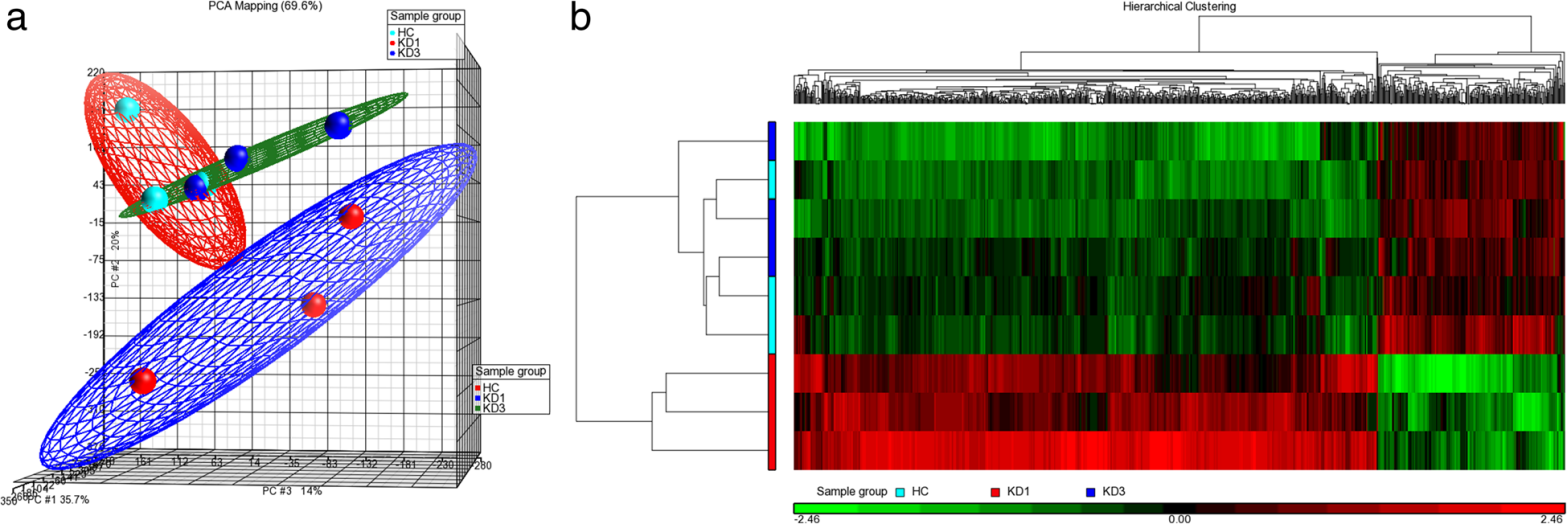
Gene expression profiles among the HC, KD1, and KD3 sets. We conducted gene expression microarray (HTA2.0) assays on three pooled HC, three pooled KD1, and three pooled KD3 samples. The generated raw data was analyzed with Partek to produce **a** a PCA plot and **b** a heat map. The heat map was plotted with the gene expression profiles of 936 genes

From the total HC, KD1, and KD3 RNA samples, we selected 18 HC, 18 KD1, and 18 KD3 ones to generate 3 HC, 3 KD1, and 3 KD3 evenly pooled samples. We then conducted the HTA 2.0 assays on the 9 pooled RNA samples (Additional file 1). The generated raw data was analyzed with Partek. Like DNA methylation, we also examined the overall gene expression patterns of the three sets with a PCA plot. As shown in Fig. 3a, the distinguishability of the three sets based on the gene expression data was not as good as that based on the DNA methylation data, especially for the HC and KD3 sets. Table 2 shows only 10 significant genes (*P* < 0.05 and expression ratio > 1.5) in the KD3 vs. HC comparison, and the union of all significant genes comprised 936 genes. Using the 936 union genes, we drew a heat map (Fig. 3b), which demonstrated that the KD3 samples were hardly distinguishable from the HC ones based on the gene expression profiles.

**Table 2:** Summary of significant genes among the comparisons

Comparison	# all genes	# up genes	# down genes
KD1 vs. HC	678	495	183
KD3 vs. HC	10	1	9
KD3 vs. KD1	810	141	669

Based on the criteria of a *p* < 0.05 and variation ratio > 1.5, we tabulated the numbers of significant genes among the three comparisons. Up gene and down gene denoted the significant upregulated and downregulated genes, respectively

### Correlation between gene expression and DNA methylation

So far, we obtained both DNA methylation and gene expression data from the HC, KD1 and KD3 samples. DNA methylation was usually thought to be negatively correlated with gene expression. The higher the CpG marker was methylated, the less abundantly the gene was expressed. However, previous studies also found positive correlations, globally or specifically [18, 19]. In addition, few studies have attempted to investigate to what extent DNA methylation on CpG marker altered gene expression. In other words, what is the global correlation pattern between DNA methylation and gene expression?

To globally and comprehensively address this question, we first constructed regulation pairs of CpG markers and genes (see the “Methods” section), followed by tabulating the variation ratios of CpG markers and genes in each comparison, e.g., KD1 vs. HC. With this approach, we could calculate the correlation coefficient between the variation ratios of gene expression and CpG marker methylation, investigating to what extent DNA methylation repressed or activated gene expression.

We first constructed random regulation pairs of CpG markers and genes by randomly assigning one CpG marker and one gene into one pair without considering whether the marker was located within the putative promoter or not. As shown in Additional file 6, the Spearman’s rank correlation coefficients of the three comparisons (random column, sub-figure a, b and c) were almost zero, reflecting pretty low correlations. Then, we considered all regulation pairs of CpG markers and genes (both significant and non-significant). We also divided the regulation pairs of CpG markers and genes into two sets, based on their genomic positions being located upstream or downstream of the TSS. As shown in Additional file 6, the upstream, downstream, and both (union of the upstream and downstream sets) columns showed that Spearman’s rho values were a little bit lower than those of the random column, reflecting slightly higher negative correlations.

**Fig. 4:**
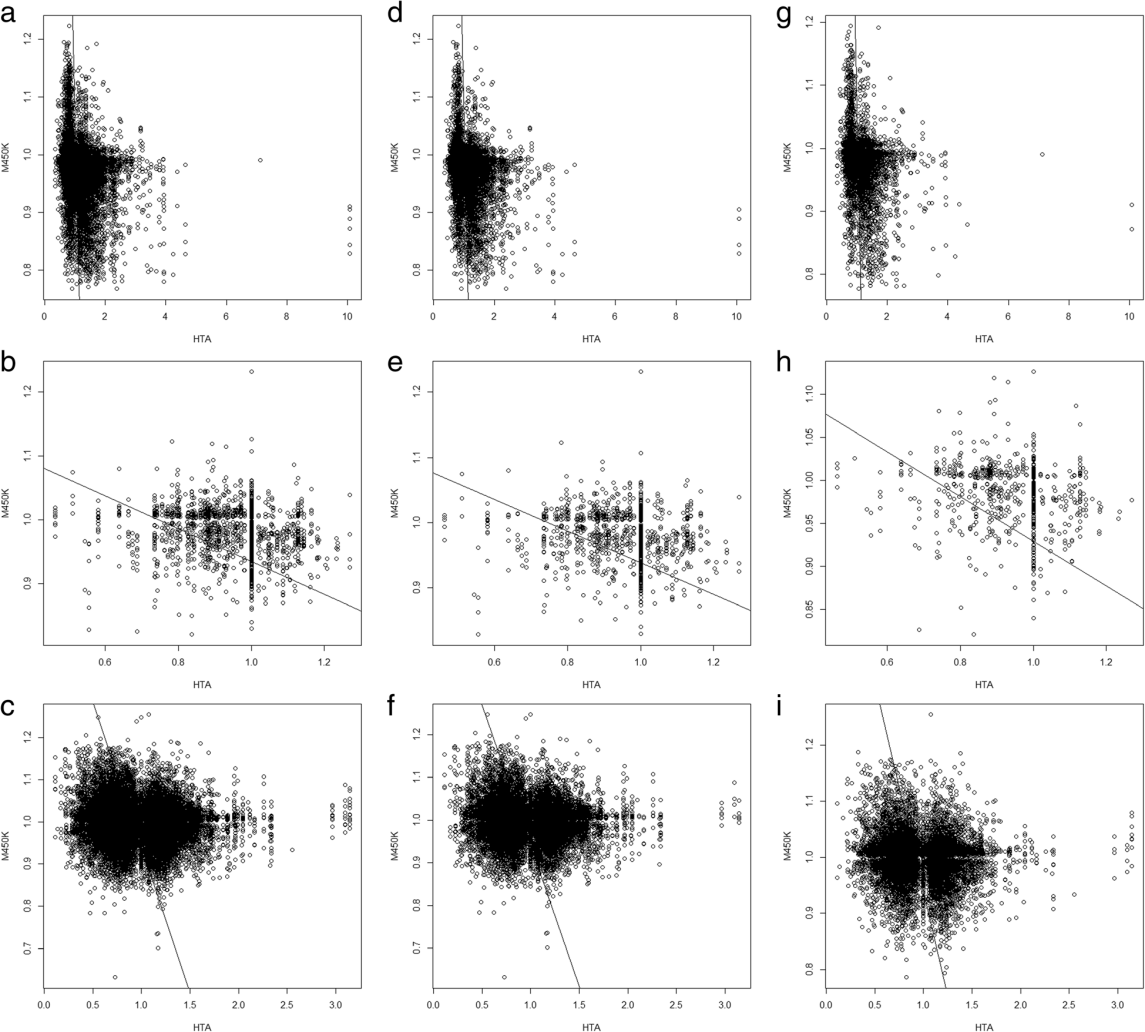
The scatter plots of gene expression variations and DNA methylation variations for CpG markers located within the putative promoters. The *X* axis presented the gene expression variation determined with the HTA2.0 assay. The *Y* axis presented the DNA methylation variation determined with the M450K assay. Each dot denoted the regulation pair of one significant gene and one significant CpG marker; only those with a *p* value < 0.05 were concerned significant. For each comparison in each column, the Spearman’s rank correlation coefficient (denoted as rho) was labeled. The correlation coefficient was calculated with the data from the full-length promoter (the Both column), in the − 5000 to − 1 bp region (the Upstream column) and the + 1 to + 3000 bp region (the Downstream column). The sample size for sub-figures **a** to **i** were in order: 28,776, 3903, 61,055, 18,068, 2318, 36,770, 10,698, 1575, and 24,285

Next, we considered only the significant CpG markers (*P* < 0.05) and the significant genes (*P* < 0.05). In other words, only significant CpG markers and genes were included to construct the regulation pairs of CpG markers and genes. As shown in Fig. 4, the upstream, downstream, and both columns showed much lower Spearman’s rho values (*P* = 0.0246, paired *t* test) than the values in Additional file 6, reflecting stronger negative correlations between the three comparisons when only significant CpG markers and genes were considered.

**Fig. 5:**
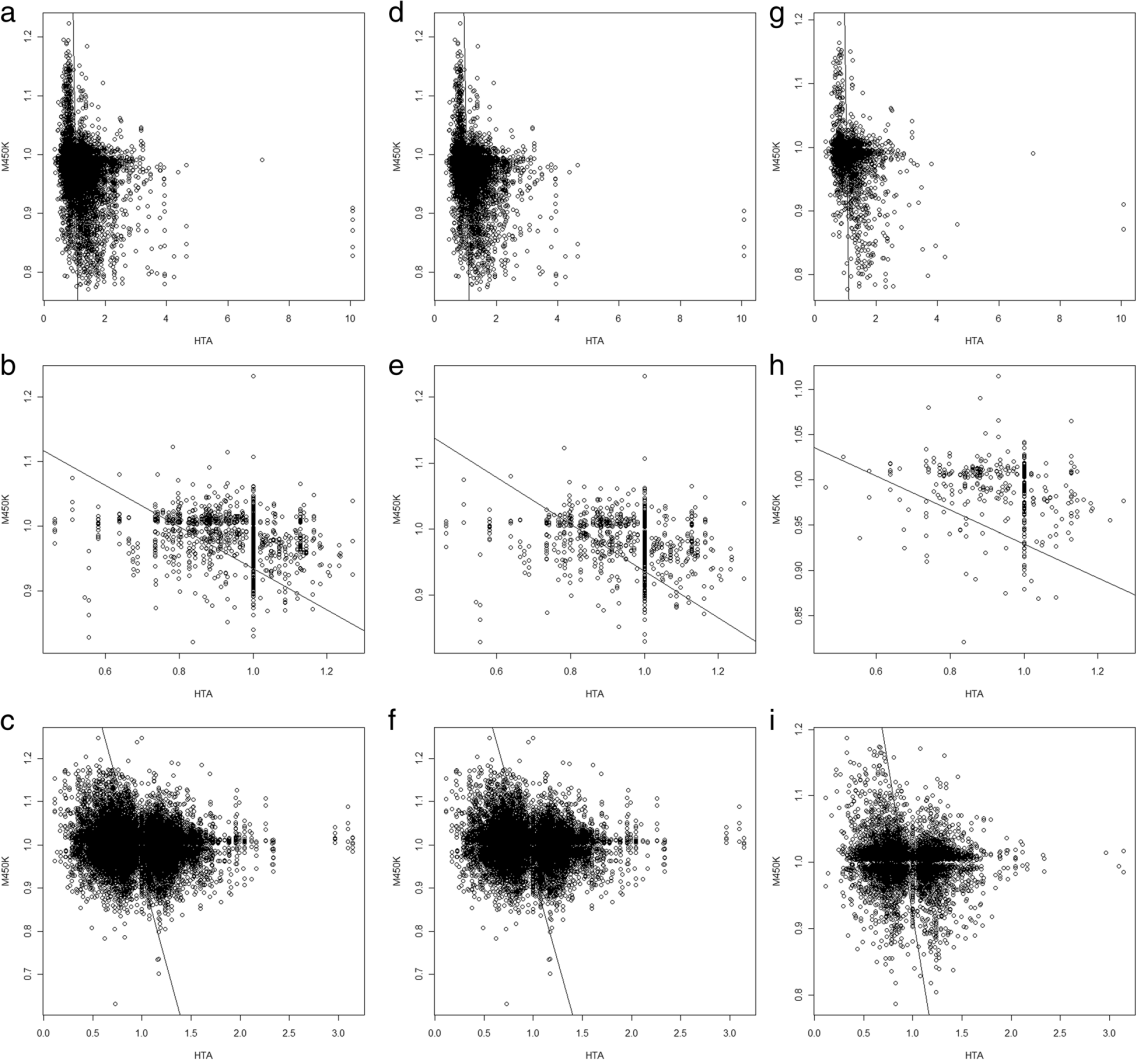
The scatter plots of gene expression variation and DNA methylation variation for CpG markers located within the core sub-regions of the putative promoters. In this figure, only the CpG markers within the core sub-region (Fig. 3) were included in this analysis. Therefore, the data presented in this figure is a subset of the one presented in Fig. 4. The Both, Upstream, and Downstream columns individually represented the − 1500 to + 500 bp, − 1500 to − 1 bp and + 1 to + 500 bp regions. The sample sizes for sub-figures **a** to **i** were in order: 17,891, 2735, 40,106, 13,298, 1868, 27,482, 4593, 867, and 12,624

Figure 2 shows that the CpG markers located within the core sub-regions of the putative promoters better regulated gene expression. So, we further performed similar analyses using only the CpG markers located within the core sub-regions (− 1500 to 500 bp). As a result, Fig. 5 shows that although not yet significant (*P* = 0.0586, paired *t* test) owing to the small sample size, 7 out of 9 comparisons (except for subfigures h and i) had stronger negative correlations than those shown in Fig. 4, which was consistent with the conclusion of Fig. 2 that the CpG makers closer to the TSSs of the transcripts better regulated gene expression.

In summary, no matter the CpG marker was located upstream or downstream of the transcript’s TSS, globally speaking, DNA methylation and gene expression maintained a modestly negative correlation, at least in the KD cases in this study.

### Perfect cases of negatively correlated genes and CpG markers

**Fig. 6:**
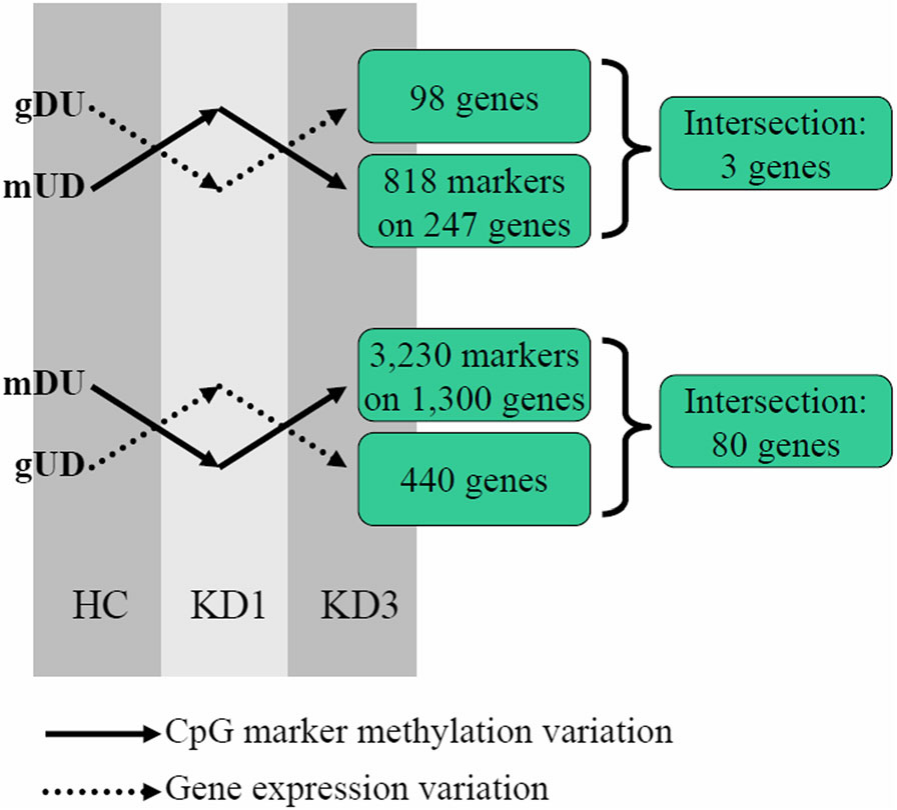
The concept of perfect cases of negatively correlated genes and CpG markers. Among the three sample sets, we were especially interested in the variations of gene and CpG markers from HC to KD1 and from KD1 to KD3. The mUD and gUD individually denoted the CpG markers and genes that were first upregulated from HC to KD1 and then downregulated from KD1 to KD3, indicating the up-then-down cases. mDU and gDU individually denoted the CpG markers and genes that were first downregulated from HC to KD1 and then upregulated from KD1 to KD3, forming the down-then-up cases. In this manner, we identified 83 genes and their promoter CpG markers that were the perfect cases of negatively correlated genes and CpG markers

In this study, we collected samples from the healthy controls (HC), patients before disease treatment (KD1), and patients after disease treatment (KD3). Therefore, we were interested in the variation profiles from HC to KD1 and from KD1 to KD3. In other words, we were interested in the genes or CpG markers that were upregulated from HC to KD1 and then downregulated from KD1 to KD3 (i.e., up-then-down cases). In addition, the down-then-up cases were also our targets. Figure 6 illustrates the perfect cases of negatively correlated genes and CpG markers. These perfect cases were composed of the up-then-down genes and the down-then-up CpG markers as well as the down-then-up genes and the up-then-down CpG markers.

Among the significant genes shown in Table 2, we identified 98 down-then-up and 440 up-then-down genes (Fig. 6). In addition, among the significant CpG markers in Table 1, we identified 3230 down-then-up and 818 up-then-down CpG markers, which were located at the promoters of 440 and 247 genes, respectively. Further intersection analyses generated 83 (80 + 3) perfect genes possessing negative correlation with CpG markers from HC to KD1 and from KD1 to KD3. Gene expression at the transcriptional level is regulated by many factors. These 83 genes were negatively correlated with DNA methylation on their promoter CpG markers not only in the HC to KD1 transition but also in the KD1 to KD3 transition. Therefore, they were the perfect targets for the further functional analysis.

### The regulatory roles of the S100A gene family

**Fig. 7:**
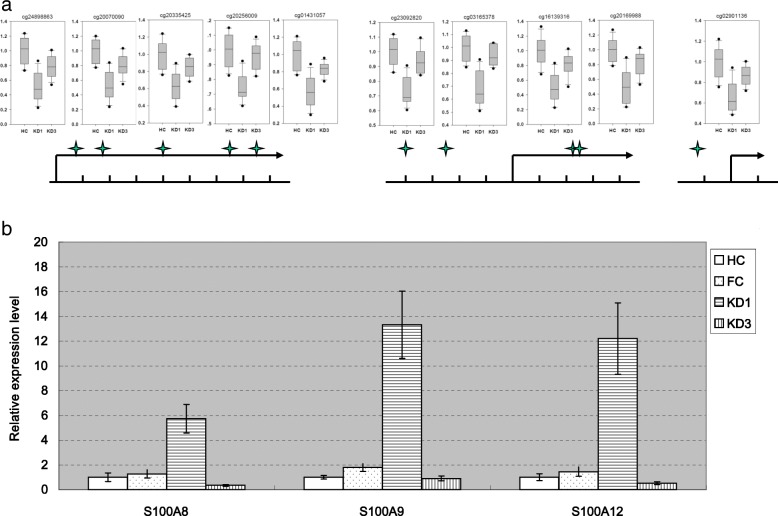
The expression variations of S100A family genes and methylation variations of the S100A-related CpG markers. **a** There were five, four, and one significant CpG markers (FDR < 0.05 and variation ratio > 1.1) located within the promoters of S100A8, S100A9, and S100A12, respectively. The locations of the ten CpG markers were indicated by the corresponding star signs. For each CpG marker, the *Y* axis of the box plot is the beta value (methylation percentage) determined with the M450K assays on 12 HC, 12 KD1, and 12 KD3 samples. **b** We used qPCR assays to detect gene expressions of S100A family genes on 24 HC, 21 FC, 17 KD1, and 18 KD3 samples. One KD1 sample failed the qPCR assay. The *Y* axis denoted the 2^−ΔΔCt^ values. The data was presented as the average ± S.D. The values of the HC set were normalized to one. * and **** denoted *p* values < 0.05 and < 0.0001, respectively

We further conducted GO analysis on the 80 genes, and the result is shown in Additional file 7. After careful inspection, we found that four out of the 80 input genes, including S100A8, S100A9, S100A12, and FCER1G, were repetitively involved in the top five GO items in terms of *p* value. Therefore, we conducted qPCR assays on the four genes and succeeded in detecting the S100A gene family, namely S100A8, S100A9, and S100A12. Figure 7a illustrates five, four, and one CpG markers on the putative promoter regions of S100A8, S100A9, and S100A12, respectively. These CpG markers were all statistically significant and were all down-then-up cases. The qPCR assays also confirmed that the S100A genes were all the up-then-down cases (Fig. 7b). In summary, in the transitions from HC to KD1 and from KD1 to KD3, the CpG markers were negatively correlated with S100A gene expressions, demonstrating epigenomic regulation abilities.

We have demonstrated a global modestly negative correlation between DNA methylation and gene expression (Figs. 4 and 5). Here, we were also interested in to what extent these 10 CpG markers regulated the S100A genes. Using the 2^−ΔΔCt^ values (Fig. 7) determined with qPCR to replace the intensity values determined with HTA2.0, we conducted similar assays. We found that the rho value between S100A8 and its promoter CpG markers was − 0.4388. And, the rho values for S100A9 and A12 were − 0.3972 and − 0.4543, respectively. Therefore, the S100A genes and their promoter CpG markers were moderately negatively correlated, indicating stronger correlations than the global profiles.

S100A8 and S100A9 are inflammatory markers that are usually highly expressed in acute and chronic inflammation. They are expressed and secreted into the plasma by neutrophils and/or monocytes, performing cytokine-like functions in inflammation [23, 24]. S100A8 and S100A9 are also involved in the pathogenesis of many diseases. They were reported to predict cardiovascular events in humans [25], to promote reticulated thrombocytosis and atherogenesis in diabetes patients [26] and to trigger inflammation, apoptosis, and tissue injury in the kidney [27]. In addition, S100A8 and S100A9 were thought to be involved in neutrophil migration in inflammatory sites [28].

In addition to the conclusions drawn from the above studies, the top GO items were also involved in leukocyte migration, neutrophil migration, and neutrophil chemotaxis (Additional file 7). Moreover, S100A8, S100A9 and S100A12 were involved in all of these GO items. Therefore, we investigated whether these S100A genes regulated neutrophil transendothelial migration, which is the causes of vascular inflammation and coronary artery aneurysm (i.e., the complication of KD). For this purpose, we conducted an in vitro leukocyte transendothelial migration (LTEM) assay. We treated neutrophil cells with the recombinant S100A family proteins and examined whether S100A treatment enhanced neutrophil transendothelial migration (migrating from the upper chamber into the lower chamber, see the “Methods” section) with an in vitro cell model, in which neutrophil cells in the lower chamber were collected and counted with flow cytometry.

**Fig. 8:**
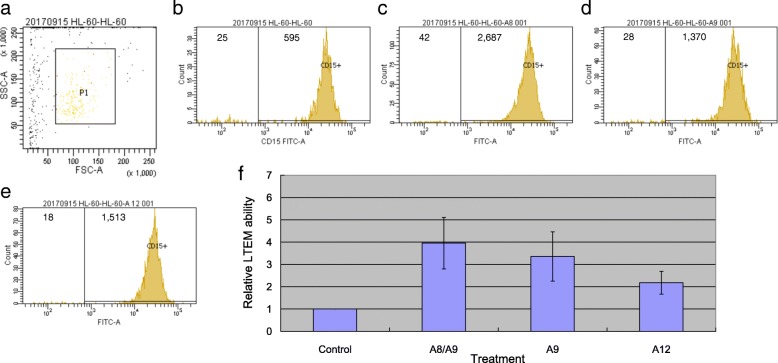
The results of flow cytometry and leukocyte transendothelial migration (LTEM) assays. We examined whether S100A family proteins influenced the LTEM ability of neutrophil cells with an in vitro cell model. **a** By specifying the FSC-A and SSC-A values, we first determined the target set of observed cells. **b**–**e** The numbers of CD15^+^ neutrophil cells with different treatments were counted. Only the value from one run of the LTEM assays was illustrated. **f** We conducted four independent runs of the LTEM assays (*n* = 4). The bars were shown as the average ± S.D. ** denoted a *p* value < 0.01

We first had non-treated neutrophil cells stained and analyzed with flow cytometry. As shown in Fig. 8a, we determined the target set of observed cells based on the specified FSC-A and SSC-A values. Then, using the same criteria, we selected the target set and counted the CD15^+^ neutrophil cells. Figure 8b shows that, without S100A treatment, 595 CD15^+^ neutrophil cells were counted. With S100A8/A9 complex, S100A9, and S100A12 treatment, 2687, 1370, and 1513 CD15^+^ neutrophil cells were counted (Fig. 8c–e), respectively. By four independent assays, compared with that of the control treatment, S100A8/A9 complex, S100A9, and S100A12 treatment all significantly promoted neutrophil cells to penetrate the endothelial layer (Fig. 8f). The ANOVA *p* value was 0.0016, and the *p* values of the individual comparisons were all less than 0.01. In addition, no significant difference was observed between any two treatments.

## Discussion

Intravenous immunoglobulin (IVIG) administration is the standard treatment for many autoimmune diseases, including idiopathic thrombocytopenic purpura, Guillain-Barré syndrome, dermatomyositis, and many others [29]. Although it is still a debate whether KD is an infectious or an autoimmune disease, IVIG is currently the most effective treatment for KD patients [13]. Based on the results from both the M27K [16] and M450K assays, IVIG administration may have a much stronger impact on DNA methylation than KD disease itself. In addition, the KD patients at the convalescent phase (KD3, 3 weeks after IVIG administration) recovered their health based on their gene expression profiles, with only 10 genes differentially expressed from the healthy control (HC) subjects. However, unlike the gene expression profiles, the DNA methylation profiles differed between the HC and KD3 sets. Actually, there are few chances to collect blood samples from KD patients without IVIG treatment at the convalescent phase. Therefore, it is difficult to determine whether such long-term variations on DNA methylation are triggered by IVIG administration or by the intrinsic immune responses against KD.

Compared with the samples from the convalescent phase, S100A8, S100A9, and S100A12 were reported to keep higher expression levels in total leukocytes from KD patients at the acute phase [30]. Moreover, S100A12 was highly correlated with the response to IVIG treatment [31], reflecting its application for monitoring the KD status [32]. Some studies showed that inflammatory cytokines were regulated through an epigenetic mechanism [33]. Our data suggested that S100A8, S100A9, and S100A12 were also regulated in this manner in KD. In spite of the previous studies, the detailed mechanisms through which S100A genes regulate the pathogenesis of KD have not yet been well studied. And, our study bridges the gap, enhancing our understanding of S100A gene family on KD pathogenesis.

Coronary artery aneurysm (CAA) is a type of vascular inflammation and the most severe complication of KD. In this study, we used an in vitro cell model to demonstrate that S100A proteins enhanced the LTEM ability of neutrophil cells, implying the regulatory mechanism in KD pathogenesis. This study showed that LTEM assay may service as an in vitro vasculitis model for KD although, so far, there is no in vitro cell model specific for KD available. S100A8 and S100A9 form a heterodimer [24, 25], and the S100A8/A9 complex is commercially available; thus, we treated neutrophil cells with the A8/A9 complex. However, although it naturally functions as a heterodimer with A8, S100A9 alone also had the potential to enhance the LTEM ability of neutrophil cells.

In this study, we found that CpG markers within the core sub-region (− 1500 to 500 bp) tended to vary more than the rest CpG markers (Fig. 2), implying that the CpG makers closer to the TSS of the transcript regulated gene expression more significantly. Actually, promoter regions of genes usually carry many functional domains, e.g., transcription factor binding sites (TFBSs), responsible for transcriptional regulations. However, promoter is a rough and ambiguous region relative to the TSS of a gene. Although long genomic fragments were defined as putative promoter regions in studies [21, 22], the functional domains confirmed by experiment or selected for experiment were usually close to the TSSs [34–36]. Such phenomenon was also consistent with our finding in Fig. 5. In summary, since the target sites of transcription factor binding and histone protein modification are close to the TSSs of genes, to perform the regulation abilities, the CpG markers close to the TSSs (− 1500 to 500 bp) tended to vary more.

The relationship between DNA methylation and gene expression may reflect the real immune response to a disease, although any part of the immune response cannot reflect the whole reaction. This study is the first to integrate a DNA methylation array with a gene expression one in KD and shows that S100A family plays important roles in the pathogenesis of KD.

## Conclusion

Although DNA methylation usually represses gene expression, several cases in which DNA methylation plays promotion roles have also been reported. In addition, globally to what extent DNA methylation represses or promotes gene expression has seldom been discussed in previous studies and has never been discussed in relation to KD. In this study, by combining DNA methylation and gene expression data, we first concluded that the CpG markers close (− 1500 bp to + 500 bp) to the TSS varied more than those located far from the TSS did. Second, we identified global modestly negative correlations between DNA methylation and gene expression regardless of whether the CpG markers were located upstream or downstream of the promoter regions. Third, we found that the S100A gene family and their promoter CpG markers were perfect cases of negative correlations. Owing to disease onset (from HC to KD1), the CpG markers were hypo-methylated, which activated S100A genes’ expressions. Owing to treatment (from KD1 to KD3), the CpG markers were hyper-methylated, which inactivated S100A genes’ expressions. Finally, we proved that S100A family proteins enhanced leukocyte transendothelial migration in KD with an in vitro cell model.

## Methods

### Subject enrollment and sample collection

We enrolled volunteer subjects from Kaohsiung Chang Gung Memorial Hospital. This study was approved by the institutional ethics board (IRB number: 201700270A3C501) and all subjects or their guardians signed the informed consent form. Whole blood samples were collected from the subjects, followed by red blood cell (RBC) lysis with RBC lysis buffer to enrich total white blood cells (WBCs). Next, we used the QIAamp® DNA Blood Mini Kit (Qiagen, CA, USA) to extract DNA and the mirVana™ miRNA Isolation Kit (Ambion, CA, USA) to extract RNA following the manufacturer’s protocols. The DNA and RNA concentrations were measured with the NanoDrop 2000 spectrophotometer (Thermo Scientific, MA, USA). All RNA samples passed the criterion of a RIN ≥ 7 assessed with the Agilent 2100 Bioanalyzer (Agilent, CA, USA).

### DNA methylation microarray assay

The extracted DNA samples were bisulfite modified with EZ DNA Methylation-Lightning™ Kit (Zymo Research, Irvine, USA). Briefly, 0.5 μg of DNA was mixed with lightning conversion reagent, followed by the thermal-cycling condition: 98 °C for 8 min, 54 °C for 60 min, and held at 4 °C. Next, the DNA samples were loaded into spin column and mixed with M-binding buffer. After centrifuge, spin column was incubated with L-desulphonation buffer at room temperature. Finally, bisulfite-modified DNA was eluted using M-elution buffer and stock at − 80 °C. After bisulfite treatment, the bisulfite-converted DNA samples were subject to genome-wide screening on DNA methylation patterns with Illumina HumanMethylation450 (M450K) BeadChip microarray assay, able to determine the methylation percentages (called beta values) of approximately 450,000 CpG markers. The microarray assays passing the quality control criteria were then analyzed with Partek, a commercial software specific for microarray data analysis.

### Gene expression microarray assay

The collected RNA samples were subject to microarray assay to determine gene expression profile. In this study, we adopted Affymetrix HTA 2.0 microarray chips for the profiling job. The RNA sample were first prepared with WT PLUS Reagent kit (Affymetrix) followed by hybridization on HTA 2.0 microarray chips. The raw data of HTA 2.0 chips were first subject to quality control examination as suggested by Affymetrix manuals. And, the chips passing the quality control criteria were then analyzed with Partek.

### Mapping CpG markers and constructing the regulation pairs of CpG marker and gene

We mapped the CpG markers back to the human genome (hg19) and examined whether they were located within the putative promoter region, ranging from 5000 bp upstream to 3000 bp downstream (− 5000 to + 3000 bp) of mRNA’s transcription start sites (TSSs) based on RefSeq 41 annotation. If so, this CpG marker was assumed to be regulating the gene, resulting in 618,621 unique regulation pairs of CpG markers and mRNAs.

Due to the existence of alternative splicing isoforms, one gene may have several mRNAs with different TSSs [37]. For example, the ABCC10 gene is located at chromosome 6 and has two alternative splicing isoforms, NM_001198934 and NM_033450, the TSSs of which are individually 43,395,292 and 43,399,489 bp. Owing to the varied TSSs and putative promoter regions, the CpG markers located at the upstream promoter of NM_001198934 could be located out of the promoter of NM_033450. Meanwhile, the CpG markers located at the downstream promoter of NM_001198934 could be located at the upstream promoter of NM_033450. Since we considered the differences in the upstream and downstream promoter regions, we enumerated all regulation pairs of CpG marker and mRNA. In addition, since we measured gene expression levels with a microarray and/or qPCR in this study, the term “mRNA” in the regulation pairs was replaced with the term “gene” for simplicity.

### Real-time quantitative polymerase chain reaction

For the real-time PCR, 0.5 μg of total RNA was reverse transcribed into cDNA using the High-Capacity cDNA Reverse Transcription Kit (Applied Biosystems, CA, USA). Next, we performed real-time quantitative PCR using the Fast SYBR® Green Master Mix system and the StepOnePlus™ System (Applied Biosystems). The sequences of the primers used were as follows:

18S: forward primer (5′-GTAACCCGTTGAACCCCATT-3′) and reverse primer (5′-CCATCCAATCGGTAGTAGCG-3′); S100A8: forward primer (5′-ACCGAGTGTCCTCAGTA-3′) and reverse primer (5′-TCTTTGTGGCTTTCTTCATGG-3′); S100A9: forward primer (5′-AACACCTTCCACCAATACT-3′) and reverse primer (5′-GCCATCAGCATGATGAACT-3′); and S100A12: forward primer (5′-CTTACAAAGGAGCTTGCAAAC-3′) and reverse primer (5′-GGTGTGGTAATGGGCAG-3′). The real-time PCR master mix was prepared as follows: 10 μl of 2X fast SYBR green master mix, 7 μl of nuclease-free water, 1 μl of cDNA, 1 μl of forward primer (10 μM), and 1 μl of reverse primer (10 μM). The default PCR thermal-cycling condition was as follows: 20 s at 95 °C and 40 cycles of 3 s at 95 °C and 30 s at 60 °C.

### Cell culture and the leukocyte transendothelial migration assay

As suggested in a previous study, we used HL-60-like neutrophil cells to conduct the migration assay [38]. The HL-60 cells (BCRC No. 60027) were induced into neutrophil-like cells by culture in Iscove’s modified Dulbecco’s medium supplemented with 20% fetal bovine serum, 4 mM l-glutamine and 1.5 g/L of sodium bicarbonate at 37 °C in a humidified 95% air/5% CO_2_ incubator. The cells were differentiated into neutrophil-like cells with the stimulus of 1.3% DMSO (Sigma-Aldrich, MO, USA). Primary human coronary endothelial cells (HCAEC, CC-2585, Clonetics, Lonza) were cultured in EBM-2 medium (CC-3156, Clonetics, Lonza) supplemented with EGM-2 MV SingleQuots (CC-4147, Clonetics, Lonza) which contains 5% FBS.

For the transendothelial migration assay, 2 × 10^5^ HCAECs were first seeded into gelatin-coated 24-well hanging inserts (also called the upper chamber, 3 μm, PET, Merck, NJ, USA) for 24 h. Then, the inserts were put into 24-well culture plates (also called lower chamber). Neutrophil-like cells were first starved for 4 h and then cultured in serum-free culture medium with 10 g/ml of S100A8/A9 (8226-S8-050, R&D), 8 g/ml of S100A9 (9254-S9-050, R&D), or 4 g/ml of S100A12 (1052-ER-050, R&D) recombinant proteins for 24 h.

On the day of the migration assay, the S100A-treated neutrophil cells were washed with serum-free culture medium. Then, 1 × 10^5^ cells were placed in the inserts, which were further moved into 24-well culture plates containing 600 μl of medium with 200 nM fMLP (Sigma-Aldrich, MO, USA) as a chemo-attractant. After 2 h of migration, the neutrophil cells penetrating the endothelial layer and migrating into the lower chamber were collected. The cells were washed with PBS and stained with CD15-FITC (340,703, BD), followed by analysis with the LSRII flow cytometer (BD Biosciences).

## Additional files

Additional file 1: Demographic data. A total of 24 healthy control subjects (HC), 21 fever control subjects (FC), and 18 KD patients participated in this study. Each HC and FC subject contributed one tube of blood sample, whereas each KD patients contributed two tubes of blood samples, one at the acute phase before IVIG treatment (KD1) and one 3 weeks after IVIG treatment (KD3). (DOC 100 kb)

Additional file 2: Manhattan plot of *p* values in the KD1 vs HC comparison. We used a Manhattan plot to demonstrate the *p* values of all CpG markers in the KD1 vs. HC comparison. In total, 482,421 CpG markers were plotted in this figure. (PNG 1154 kb)

Additional file 3: Manhattan plot of *p* values in the KD3 vs HC comparison. We used a Manhattan plot to demonstrate the *p* values of all CpG markers in the KD3 vs. HC comparison. In total, 482,421 CpG markers were plotted in this figure. (PNG 1025 kb)

Additional file 4: Manhattan plot of *p* values in the KD3 vs KD1 comparison. We used a Manhattan plot to demonstrate the *p* values of all CpG markers in the KD3 vs. KD1 comparison. In total, 482,421 CpG markers were plotted in this figure. (PNG 1078 kb)

Additional file 5: Methylation variations of all CpG markers within the putative promoter regions. By referring to the RefSeq 41 annotation, we can determine a CpG marker’s distances to the transcription start site (TSS) of a gene’ transcript. Then, we can also determine the relative locations of CpG markers within the putative promoter regions, which are the genomic regions ranging from the − 5000 bp to + 3000 bp of a transcript’s TSS. (a, b, c) For each CpG marker, the *X* and *Y* axes denoted its methylation variation and its distance to the TSS, respectively. Using the two arrows, the promoter was split into three sub-regions, the left, the core, and the right sub-regions. The sample sizes for all sub-figures were 618,620, 618,553, and 618,553, respectively. (TIF 12711 kb)

Additional file 6: The scatter plots of all gene expression variations and all DNA methylation variations for CpG markers located within the putative promoters. Each dot denoted a regulation pair of one CpG marker and one gene, significant and non-significant. Since there were around 618,620 regulation pairs of CpG markers and genes in Additional file 5, we constructed the same number of random regulation pairs in the “Random” column. The sample sizes for the Both column were all 577,657; the sample sizes for Upstream column were all 347,878; the sample sizes for Downstream column were all 229,779. (TIF 785 kb)

Additional file 7: GO analysis results. We had the 80 genes analyzed with GO by mapping the genes to GO data set (Gene Ontology-Homo sapiens-2010-04-29). (XLS 344 kb)

## Abbreviations

CAA: Coronary artery aneurysm
KD: Kawasaki disease
LTEM: Leukocyte trans-endothelium migration
TSS: Transcription start site
