# Supplementary material for: Multiomics analyses identified epigenetic modulation of the S100A gene family in Kawasaki disease and their significant involvement in neutrophil transendothelial migration
Source: Clin Epigenetics. 2018 Nov 1;10:135. doi: 10.1186/s13148-018-0557-1 (PMC6211403; doi:10.1186/s13148-018-0557-1)
Supplement: Supplementary file 1 — Demographic data. A total of 24 healthy control subjects (HC), 21 fever control subjects (FC), and 18 KD patients participated in this study. Each HC and FC subject contributed one tube of blood sample, whereas each KD patients contributed two tubes of blood samples, one at the acute phase before IVIG treatment (KD1) and one 3 weeks after IVIG treatment (KD3). (DOC 100 kb) [file 13148_2018_557_MOESM1_ESM.doc]

**Additional file 1. Demographic data.** 24 healthy control subjects (HC), 21 fever control subjects (FC) and 18 KD patients participated in this study. Each HC and FC subject contributed one tube of blood sample; while, each KD patients contributed two tubes of blood samples, one at the acute phase before IVIG treatment (KD1) and one three weeks after IVIG treatment (KD3).

| Subject ID | Age | Gender | Diagnosis | Involved assays |
| --- | --- | --- | --- | --- |
| HC01 | 2.9 | F | HC | HTA, M450K, qPCR |
| HC02 | 1.1 | M | HC | HTA, M450K, qPCR |
| HC03 | 1.9 | M | HC | HTA, M450K, qPCR |
| HC04 | 3 | F | HC | HTA, M450K, qPCR |
| HC05 | 2.1 | F | HC | HTA, M450K, qPCR |
| HC06 | 2 | F | HC | HTA, M450K, qPCR |
| HC07 | 2.1 | F | HC | HTA, M450K, qPCR |
| HC08 | 2 | M | HC | HTA, M450K, qPCR |
| HC09 | 3 | F | HC | HTA, M450K, qPCR |
| HC10 | 2 | M | HC | HTA, M450K, qPCR |
| HC11 | 2 | M | HC | HTA, M450K, qPCR |
| HC12 | 2.5 | M | HC | HTA, M450K, qPCR |
| HC13 | 0.6 | M | HC | HTA, qPCR |
| HC14 | 0.74 | M | HC | HTA, qPCR |
| HC15 | 2.6 | F | HC | HTA, qPCR |
| HC16 | 7.6 | M | HC | HTA, qPCR |
| HC17 | 0.5 | M | HC | HTA, qPCR |
| HC18 | 0.8 | F | HC | HTA, qPCR |
| HC19 | 4.38 | F | HC | qPCR |
| HC20 | 10.1 | F | HC | qPCR |
| HC21 | 3.03 | F | HC | qPCR |
| HC22 | 4.71 | M | HC | qPCR |
| HC23 | 4.24 | F | HC | qPCR |
| HC24 | 2.53 | F | HC | qPCR |
| FC01 | 5.76 | M | FC: Acute tonsillitis | qPCR |
| FC02 | 3.39 | F | FC: Acute sinusitis | qPCR |
| FC03 | 6.04 | M | FC: Bronchopneumonia | qPCR |
| FC04 | 5.99 | M | FC: Influenza | qPCR |
| FC05 | 0.89 | F | FC: Gastroenteritis | qPCR |
| FC06 | 10.34 | M | FC: Pneumonia | qPCR |
| FC07 | 0.84 | M | FC: Lymphadenitis | qPCR |
| FC08 | 6.39 | F | FC: Acute tonsillitis | qPCR |
| FC09 | 1.41 | M | FC: Gastroenteritis | qPCR |
| FC10 | 0.97 | M | FC: Acute tonsillitis | qPCR |
| FC11 | 4.11 | F | FC: Acute tonsillitis | qPCR |
| FC12 | 1.78 | F | FC: Bronchopneumonia | qPCR |
| FC13 | 5.48 | F | FC: Acute tonsillitis | qPCR |
| FC14 | 3.65 | M | FC: Bronchopneumonia | qPCR |
| FC15 | 5.33 | M | FC: Influenza | qPCR |
| FC16 | 3.83 | M | FC: Acute tonsillitis | qPCR |
| FC17 | 11.47 | M | FC: Acute tonsillitis | qPCR |
| FC18 | 4.88 | F | FC: Cellulitis | qPCR |
| FC19 | 3.45 | M | FC: Gastroenteritis | qPCR |
| FC20 | 3.5 | M | FC: Gastroenteritis | qPCR |
| FC21 | 0.21 | M | FC: Young infant fever | qPCR |
| KD01 | 2.3 | M | KD | HTA, M450K, qPCR |
| KD02 | 1 | F | KD | HTA, M450K, qPCR |
| KD03 | 2.4 | F | KD | HTA, M450K, qPCR |
| KD04 | 2.7 | M | KD | HTA, M450K, qPCR |
| KD05 | 1 | M | KD | HTA, M450K, qPCR |
| KD06 | 1 | F | KD | HTA, M450K, qPCR |
| KD07 | 0.3 | M | KD | HTA, M450K, qPCR |
| KD08 | 1.8 | M | KD | HTA, M450K, qPCR |
| KD09 | 0.6 | F | KD | HTA, M450K, qPCR |
| KD10 | 2.6 | F | KD | HTA, M450K, qPCR |
| KD11 | 2.7 | F | KD | HTA, M450K, qPCR |
| KD12 | 0.3 | M | KD | HTA, M450K, qPCR |
| KD13 | 1 | M | KD | HTA, qPCR |
| KD14 | 1.5 | M | KD | HTA, qPCR |
| KD15 | 1.3 | M | KD | HTA, qPCR |
| KD16 | 0.8 | M | KD | HTA, qPCR |
| KD17 | 2.6 | M | KD | HTA, qPCR |
| KD18 | 1 | M | KD | HTA, qPCR |
