# Supplementary figures and images for: Multiomics analyses identified epigenetic modulation of the S100A gene family in Kawasaki disease and their significant involvement in neutrophil transendothelial migration
Source: Clin Epigenetics. 2018 Nov 1;10:135. doi: 10.1186/s13148-018-0557-1 (PMC6211403; doi:10.1186/s13148-018-0557-1)

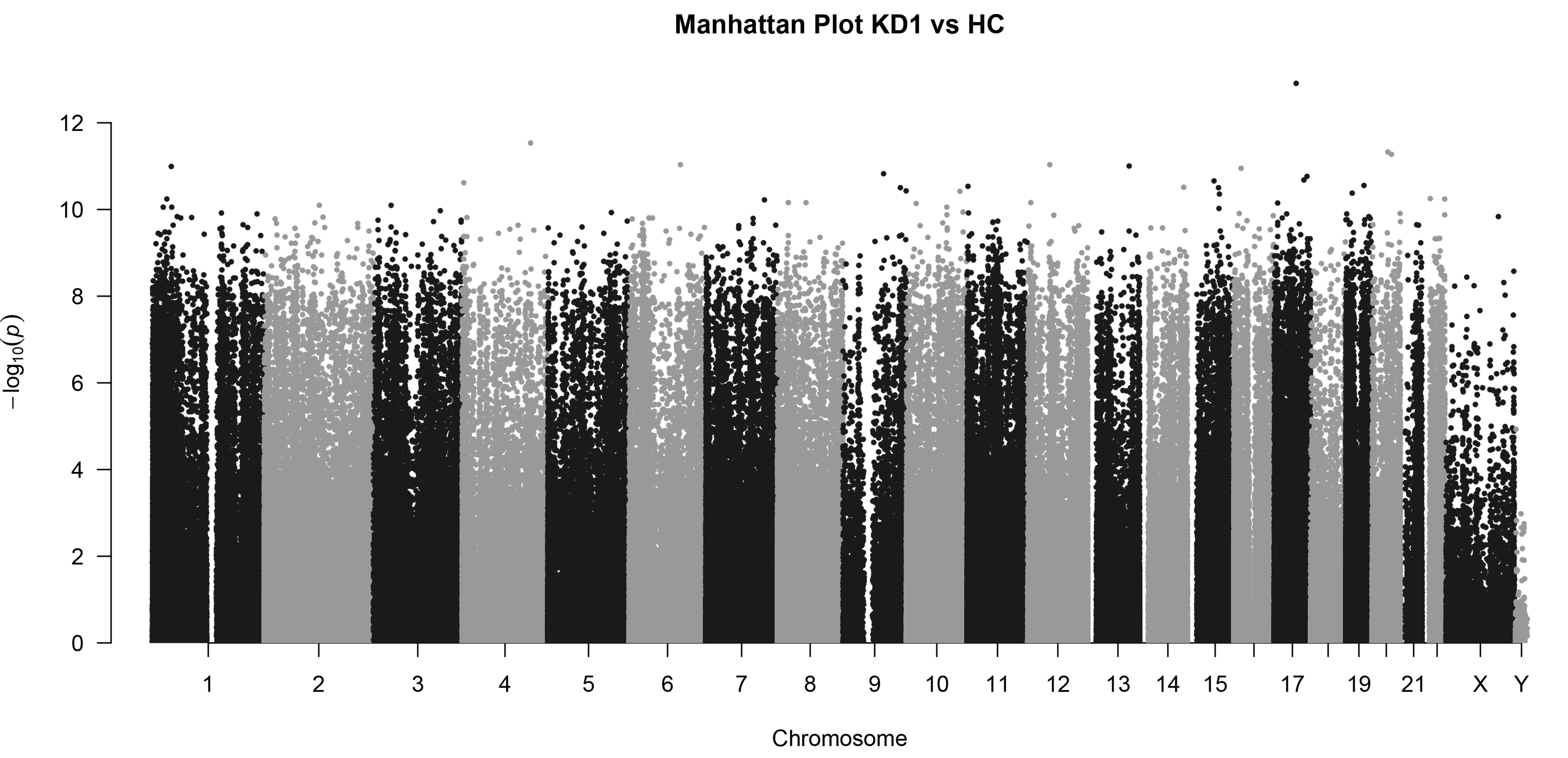

Supplement: Supplementary file 2 — Manhattan plot of p values in the KD1 vs HC comparison. We used a Manhattan plot to demonstrate the p values of all CpG markers in the KD1 vs. HC comparison. In total, 482,421 CpG markers were plotted in this figure. (PNG 1154 kb) [file 13148_2018_557_MOESM2_ESM.png]

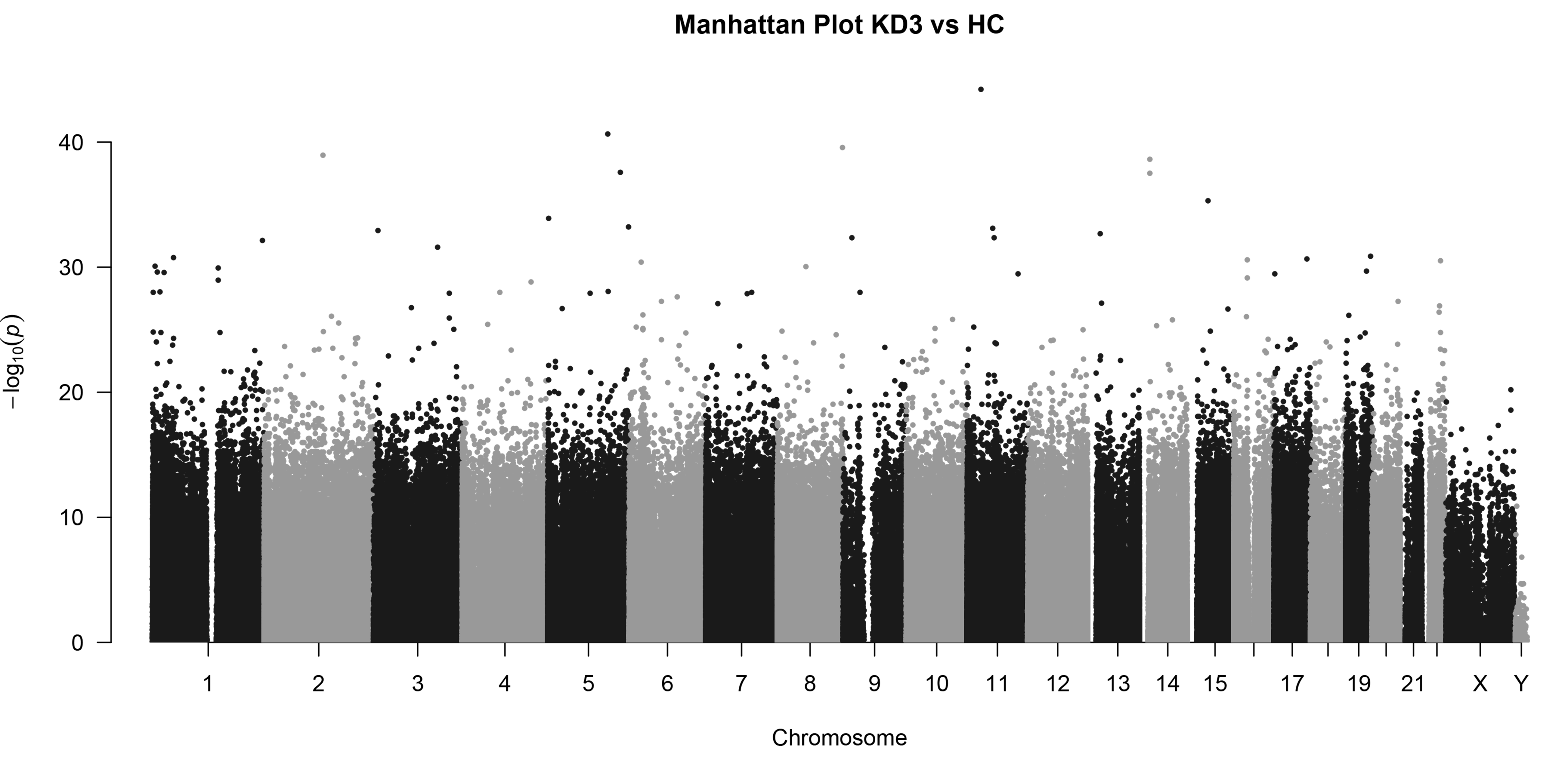

Supplement: Supplementary file 3 — Manhattan plot of p values in the KD3 vs HC comparison. We used a Manhattan plot to demonstrate the p values of all CpG markers in the KD3 vs. HC comparison. In total, 482,421 CpG markers were plotted in this figure. (PNG 1025 kb) [file 13148_2018_557_MOESM3_ESM.png]

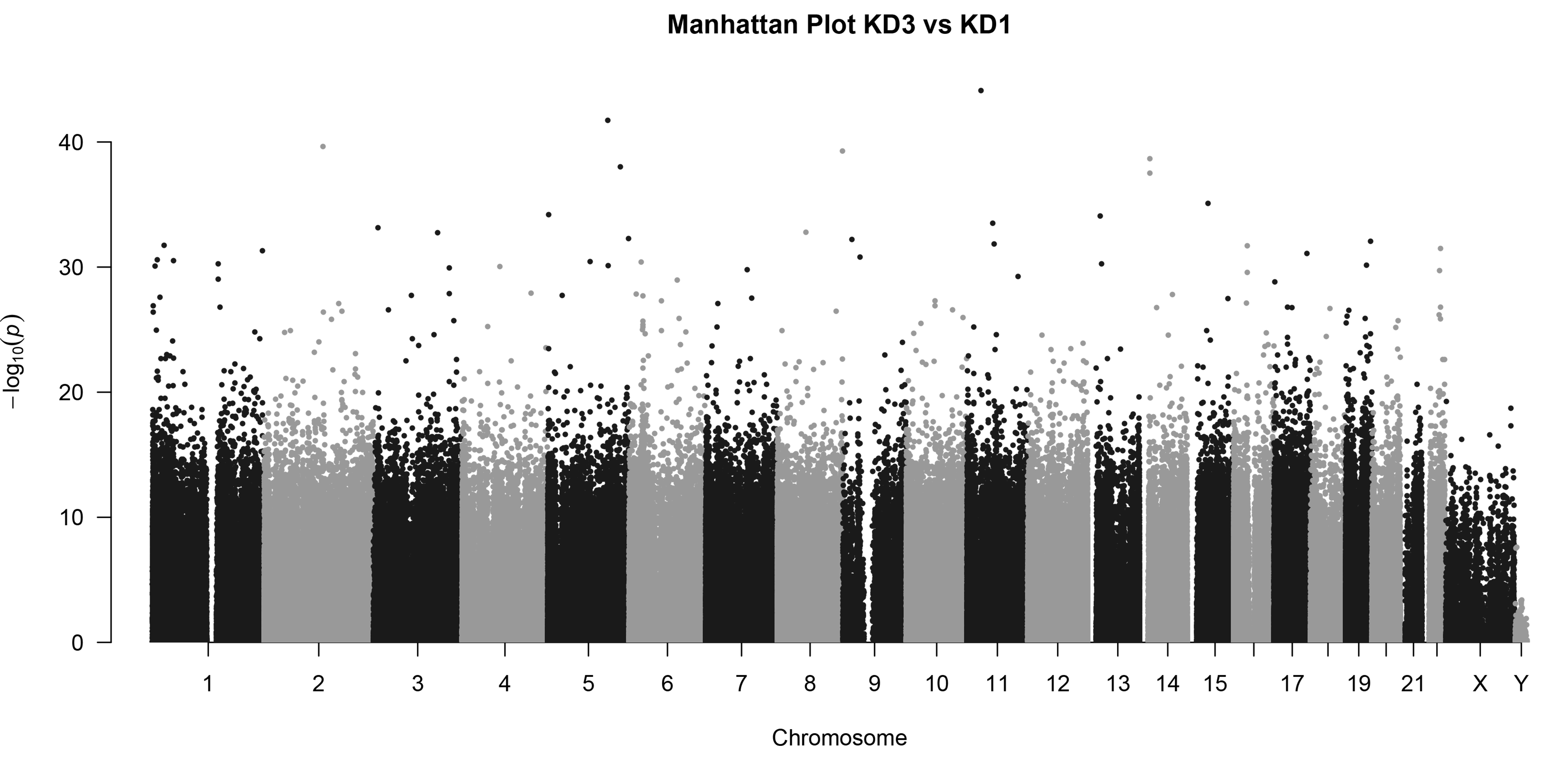

Supplement: Supplementary file 4 — Manhattan plot of p values in the KD3 vs KD1 comparison. We used a Manhattan plot to demonstrate the p values of all CpG markers in the KD3 vs. KD1 comparison. In total, 482,421 CpG markers were plotted in this figure. (PNG 1078 kb) [file 13148_2018_557_MOESM4_ESM.png]

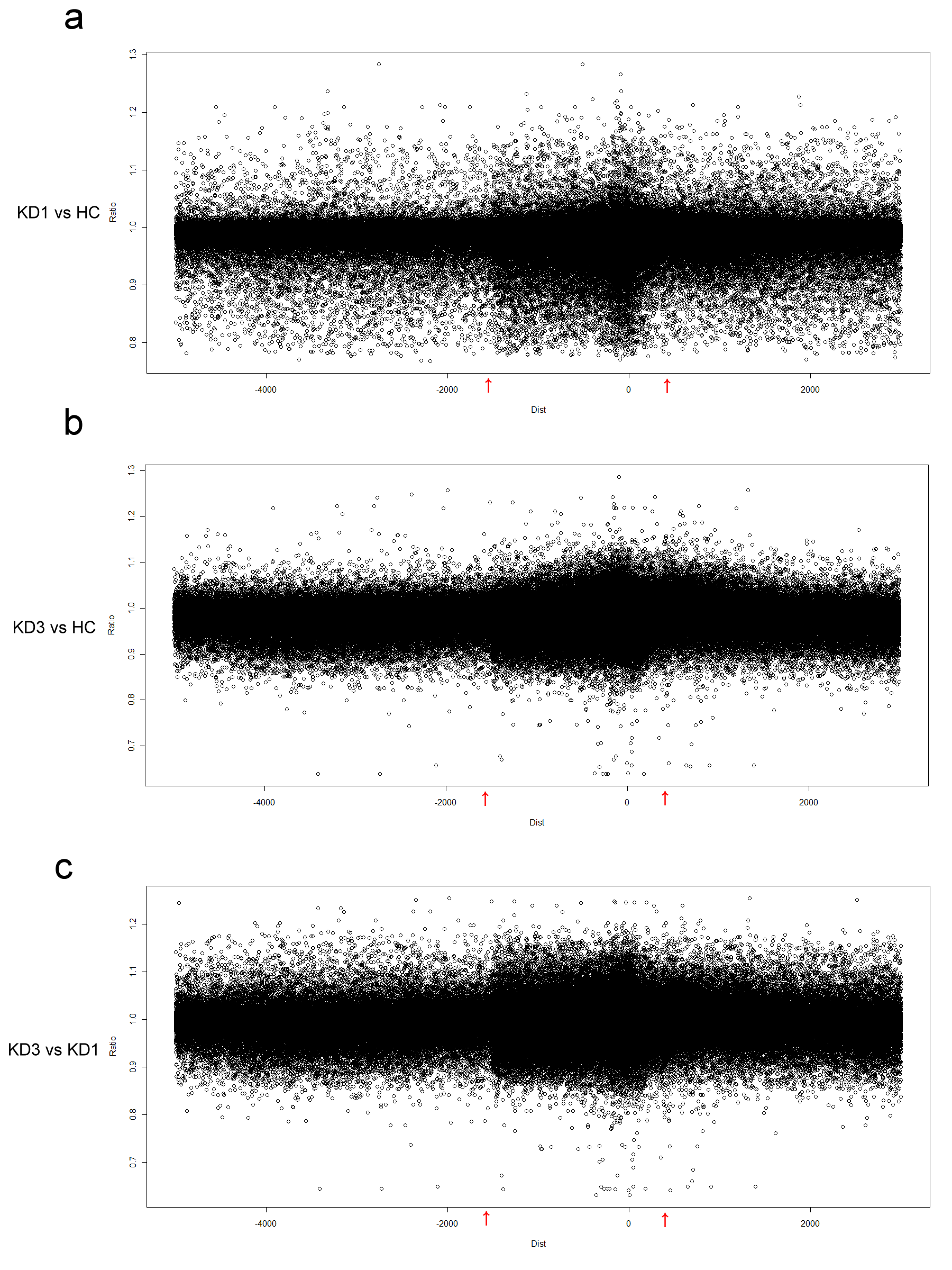

Supplement: Supplementary file 5 — Methylation variations of all CpG markers within the putative promoter regions. By referring to the RefSeq 41 annotation, we can determine a CpG marker’s distances to the transcription start site (TSS) of a gene’ transcript. Then, we can also determine the relative locations of CpG markers within the putative promoter regions, which are the genomic regions ranging from the − 5000 bp to + 3000 bp of a transcript’s TSS. (a, b, c) For each CpG marker, the X and Y axes denoted its methylation variation and its distance to the TSS, respectively. Using the two arrows, the promoter was split into three sub-regions, the left, the core, and the right sub-regions. The sample sizes for all sub-figures were 618,620, 618,553, and 618,553, respectively. (TIF 12711 kb) [file 13148_2018_557_MOESM5_ESM.tif]

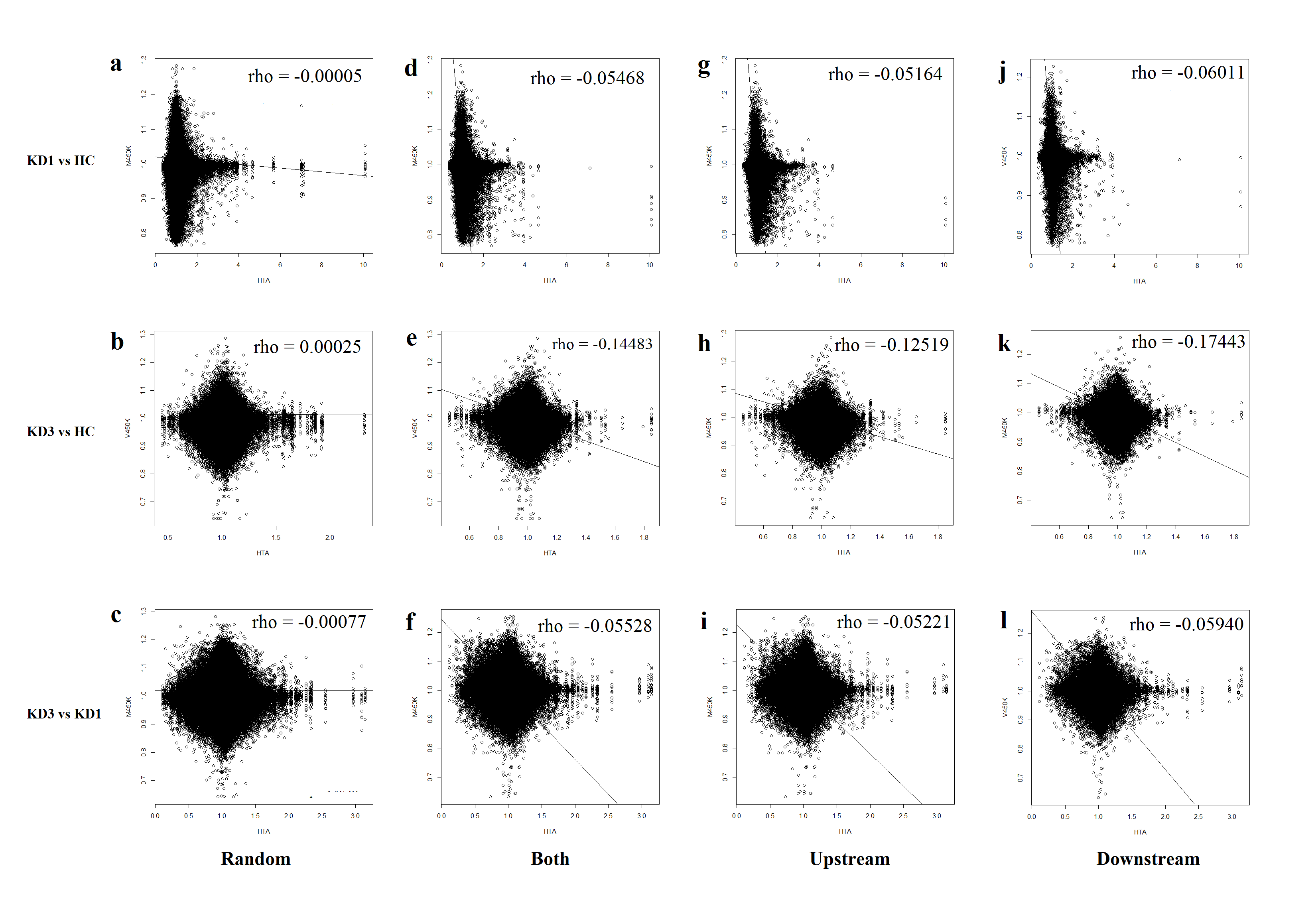

Supplement: Supplementary file 6 — The scatter plots of all gene expression variations and all DNA methylation variations for CpG markers located within the putative promoters. Each dot denoted a regulation pair of one CpG marker and one gene, significant and non-significant. Since there were around 618,620 regulation pairs of CpG markers and genes in Additional file 5, we constructed the same number of random regulation pairs in the “Random” column. The sample sizes for the Both column were all 577,657; the sample sizes for Upstream column were all 347,878; the sample sizes for Downstream column were all 229,779. (TIF 785 kb) [file 13148_2018_557_MOESM6_ESM.tif]
